# A synergistic alliance between nematophagous fungi and organic matter against plant‐parasitic nematodes: a systematic review

**DOI:** 10.1002/ps.70949

**Published:** 2026-05-21

**Authors:** Ndivhuwo Ramatsitsi, Alen Manyevere, Admire Rukudzo Dzvene

**Affiliations:** ^1^ Department of Agronomy University of Fort Hare Alice South Africa; ^2^ Centre for Global Change, University of Fort Hare Alice South Africa

**Keywords:** biocontrol agents, green technology, nematode suppression, rhizosphere, soil amendments, soil health

## Abstract

Nematophagous fungi (NF) are essential biological control agents for managing plant‐parasitic nematode (PPN) populations, which cause significant global crop losses. Through its effects on soil microbial activity and physicochemical properties, soil organic matter (OM) improves NF survival, colonisation, and functioning. This systematic review critically synthesised evidence on the potential synergistic interaction between NF and OM (i.e., enhanced nematode suppression when applied together compared to individually) for sustainable nematode suppression. Using PRISMA guidelines, studies were systematically screened based on predefined inclusion criteria, resulting in 33 studies being selected from an initial pool of 603 publications across major databases. According to our analysis, *Trichoderma* species dominate in commercial biocontrol applications, whereas *Paecilomyces* species are the most reported NF in OM‐enhanced nematode management. Even though they are recently identified, other genera, including *Talaromyces*, *Pochonia*, and *Arthrobotrys*, are seldom used under field conditions. Both NF and OM have been shown to jointly promote improved rhizosphere colonisation, enzymatic activity, and nematode management, with implications for soil health and functionality. The type and quantity of OM, the compatibility of fungal species, soil conditions, and the timing of amendments incorporation are important modifiers of this synergy. NF also function as multifunctional agents in agroecosystems by contributing to nitrogen cycling, OM decomposition, and stability in addition to biocontrol. Despite these advantages, the molecular mechanisms, field‐scale efficacy, and economic viability of NF–OM combinations across different agroecological settings remain insufficiently characterised. This study highlights the potential of NF–OM integration as a promising approach for sustainable nematode management, with possible relevance to climate‐smart agricultural practices, as well as potential implications for sustainable pest control and soil restoration with relevance to Sustainable Development Goals (SDGs) 2 (Zero Hunger), 12 (Responsible Consumption and Production), 13 (Climate Action), and 15 (Life on Land). © 2026 The Author(s). *Pest Management Science* published by John Wiley & Sons Ltd on behalf of Society of Chemical Industry.

## Graphical abstract

Certain beneficial fungi are widely used to control harmful nematodes in crops, especially when combined with organic matter. Together, they improve soil health, support plant growth, and enhance nutrient cycling. This natural partnership offers a promising, environmentally friendly approach to strengthening sustainable agriculture and reducing reliance on chemical controls.

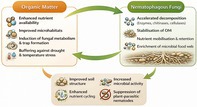

## 1 INTRODUCTION

Agricultural pests, including plant‐parasitic nematodes (PPNs), threaten global food security by causing substantial crop losses globally, with some estimates suggesting losses of up to 40% depending on crop type, region, and production system.^1^ They are among the most significant groups of organisms that live in the rhizosphere, substantially contributing to the development and yield of plants. These soilborne organisms are among the most economically significant agricultural pests, causing an estimated yearly loss of USD 173 billion worldwide.^2^ According to Molloy *et al*.,^3^ PPNs are obligate biotrophs that reconfigure host plant cells through complex host–pathogen interaction. PPNs further serve as vectors and predispose the plants to secondary microbiological (bacterial and fungal) infections.^4^ Management of PPNs historically depended on highly effective synthetic chemical nematicides. However, increasing environmental and regulatory concerns have led to the restriction and phased withdrawal of several organophosphate‐ and carbamate‐based chemical nematicides in many regions.^5^ Such restrictions resulted in significant financial losses and a significant gap in crop production and protection. It is anticipated that yield losses from PPNs would substantially rise considering climate change and the elimination of certain nematicides due to their detrimental effects on the environment.^1^ These challenges highlight the need for sustainable and integrative nematode management strategies.^6^, ^7^ One of these approaches includes the use of biocontrol agents (BCAs).^8^ The rich ecological diversity of soil microbes is a natural endowment that can be harnessed in pest management without exacerbating the already growing environmental pollution.^9^, ^10^

For cost‐effective PPN management, nematophagous fungi (NF) present viable substitutes and an environmentally friendly strategy. It has been reported that a variety of rhizospheric fungi, including *Trichoderma* and *Paecilomyces lilacinus*, efficiently manage PPNs through variety of mechanisms, such as preventing egg hatching and eliminating females and second‐stage juvenile nematodes in the soil. These NF may inhibit PPNs directly or indirectly by producing lytic enzymes, antibiotics, volatile compounds, and toxic metabolites, as well as by competing for nutrients and niche. Rather than acting independently, these mechanisms typically operate in combination, with their effectiveness varying depending on the fungal species, target nematode, and environmental conditions. Incorporation of organic matter (OM) into the soil provides multiple benefits, including serving as a carbon and nutrient source that supports NF growth and activity.^11^ The efficacy and survival of NF depend on several parameters, including environmental factors such as OM, pH, and carbon dioxide. OM promotes the growth of beneficial bacteria, fungi, and nematodes that are antagonistic to PPNs. Additionally, OM enhances soil fertility and structure, including improvements in nutrient availability, aggregate stability, and water retention, thereby rendering the environment less conducive to PPNs proliferation.^12^, ^13^ The combined use of NF and OM has increasingly been explored as an integrated approach to improving biological control of PPNs. In this context, ‘synergy’ refers to interactions where the combined application of NF and OM results in enhanced suppression of PPNs or improved soil functioning beyond the effects observed when each component is applied independently. Although the individual roles of NF and organic amendments in suppressing PPNs are well documented, their combined effects remain inconsistently characterised across studies. In particular, there is limited synthesis of how different types of OM influence fungal efficacy, the conditions under which such interactions occur, and the extent to which they contribute to sustained nematode suppression under field‐relevant conditions. This gap limits the development of optimised, biologically based management strategies.

Accordingly, the purpose of this review was to answer the following questions: (i) How do soil amendments improve NF effectiveness against PPNs? (ii) What components of OM enhance NF activity against PPNs? (iii) Does integrating OM with NF improve long‐term soil health and fertility? and (iv) How do NF contribute to the decomposition, stabilisation or nutrient cycling of added OM? This paper therefore seeks to investigate the effect of rhizosphere amendments on the efficacy of NF against economically important PPN species across their host systems.

## 2 REVIEW METHODOLOGY

### 2.1 Search strategy

The search, selection, and collection of pertinent articles were done in a methodical way using the Preferred Reporting Items for Systematic Reviews and Meta‐Analyses (PRISMA) flow‐diagram.^14^ A variety of search engines, including ScienceDirect (https://www.sciencedirect.com/), SCOPUS (https://www.scopus.com), and Google Scholar (https://scholar.google.com/), were used for collecting research articles on the interactions between various NF species and the effects of various soil OM on PPNs. Review articles and book chapters were consulted to provide conceptual background and contextual support; however, only primary experimental studies were included in the data extraction and quantitative synthesis. Both common and scientific names were utilised for specialised search queries for fungal species linked to PPNs. The phrases ‘plant‐parasitic nematodes and crop yield loss’, ‘nematophagous fungi and nematode suppression’, and ‘organic amendments and nematophagous fungi efficacy’ were also searched to compile a collection of research on this topic. Searches were limited to the English language, and although recent publications from 2020 to 2025 were prioritised, there were no publication date constraints. To enhance transparency and reproducibility, the final search string used in Scopus was as follows: TITLE‐ABS‐KEY [(‘nematophagous fungi’ OR ‘nematode‐trapping fungi’ OR ‘egg‐parasitic fungi’ OR ‘fungal biocontrol agents’) AND (‘plant‐parasitic nematodes’ OR ‘root‐knot nematodes’ OR *Meloidogyne* OR *Heterodera* OR *Rotylenchulus*) AND (‘organic matter’ OR ‘organic amendments’ OR compost OR vermicompost OR manure OR ‘soil amendments’ OR biochar)]. The search was conducted across titles, abstracts, and keywords. This search string was adapted for use in other databases, including ScienceDirect and Google Scholar, with minor modifications to syntax where required.

### 2.2 Article assessment and selection

The publication title was the focus of the main literature search phrases, which produced a first set of 603 publications. The keywords ‘plant‐parasitic nematodes’, ‘fungal biocontrol agents’, and ‘soil amendments’ were included in the search criteria. The papers were subjected to additional screening to identify original research containing quantitative and qualitative assessment of the following response variables: (i) NF effects on PPNs and (ii) soil amendments influence on the effectiveness of NF against PPNs. In addition, studies including one or more PPN species with a non‐inoculated control were included in the review. Given that the experimental research publications cited were cross‐referenced and evaluated for inclusion eligibility, reviews and book chapters were also included for the analyses.

The following selection criteria were applied to the retrieved articles: studies that found NF species whether examined independently or in combination with soil amendments, studies that calculated or assessed the population densities of one or more PPNs, and studies that involved fieldwork or research under controlled conditions. Both quantitative and qualitative information on the experiment's location, PPNs species, NF species, soil amendments, experimental conditions, variables examined, and experimental factor amounts was collected from the carefully selected publications. Studies were excluded if they did not involve NF, did not assess PPNs, lacked measurable outcomes related to nematode suppression, were not primary research articles (e.g., opinion pieces or editorials); or did not provide sufficient methodological detail for data extraction. The literature was reduced to 160 peer‐reviewed publications published between 1966 and 2025 after additional screening removed duplicates and studies without a control. Only the abstracts of papers of relevance were translated, notwithstanding efforts to get translated versions of non‐English articles, which may have introduced language bias and affected the comprehensiveness of the review. These publications were subsequently disregarded since neither the kind of soil amendment employed nor the species of nematodes were mentioned in the abstracts. These steps and standards were followed in our thorough assessment, and Table 1 provides an overview of the investigations.

**Table 1:** Eligibility criteria defining the inclusion and exclusion of studies evaluating nematophagous fungi and organic amendments for plant‐parasitic nematode management

Criteria	Exclusion	Inclusion
Article type	Non‐peer‐reviewed research articles, surveys, editorials, commentary, letters, and notes	Peer‐reviewed research and review papers
Nematode genus	Studies that do not focus on nematodes	Studies that focus on nematodes
Language	Non‐English	English
Publication date	None	Not applicable
Plant parameters	Studies that did not assess at least one plant parameter	Studies that assessed at least one plant parameter
Nematode parameters	Studies that did not assess at least one plant‐parasitic nematode metric	Studies that assessed at least one plant‐parasitic nematode metric
Soil amendments	Absence of soil amendments	Presence of soil amendments

### 2.3 Risk of bias assessment

To evaluate the methodological quality and potential bias of included studies, the Crowe Critical Appraisal Tool^15^ was applied across the following domains: preliminaries, introduction, study design, sampling, data collection, results, and discussion. Particular attention was paid to risks associated with study design, including insufficiently described experimental protocols, lack of control treatments, or poorly justified use of specific fungal strains and soil amendments. Sampling bias was noted in studies where site selection, replication, or soil variability were inadequately addressed. Inconsistencies in data collection methods, especially in measuring nematodes suppression or fungal colonisation, posed risks to internal validity. Interpretation bias was identified in cases where confounding variables (e.g., study duration or conditions) were not sufficiently controlled or discussed. The methodological quality of included studies was assessed using a simplified scoring framework informed by the Crowe Critical Appraisal Tool. The Crowe Critical Appraisal Tool framework enabled a structured and transparent appraisal of study quality, helping to distinguish between higher and lower confidence evidence in assessing the synergistic role of NF and OM in managing PPNs Each study was evaluated against five criteria: (i) clearly described experimental conditions (e.g., field, greenhouse, or net‐house), (ii) presence of a control or comparator treatment, (iii) reporting of quantitative outcomes (e.g., percentage nematode suppression, growth parameters, or infection indices), (iv) specification of study duration, and (v) relevance to field or applied conditions. Each criterion was assigned one point, resulting in a total quality score ranging from 1 to 5, with higher scores indicating more robust methodological reporting.

### 2.4 Data extraction and analysis

**Figure 1:**
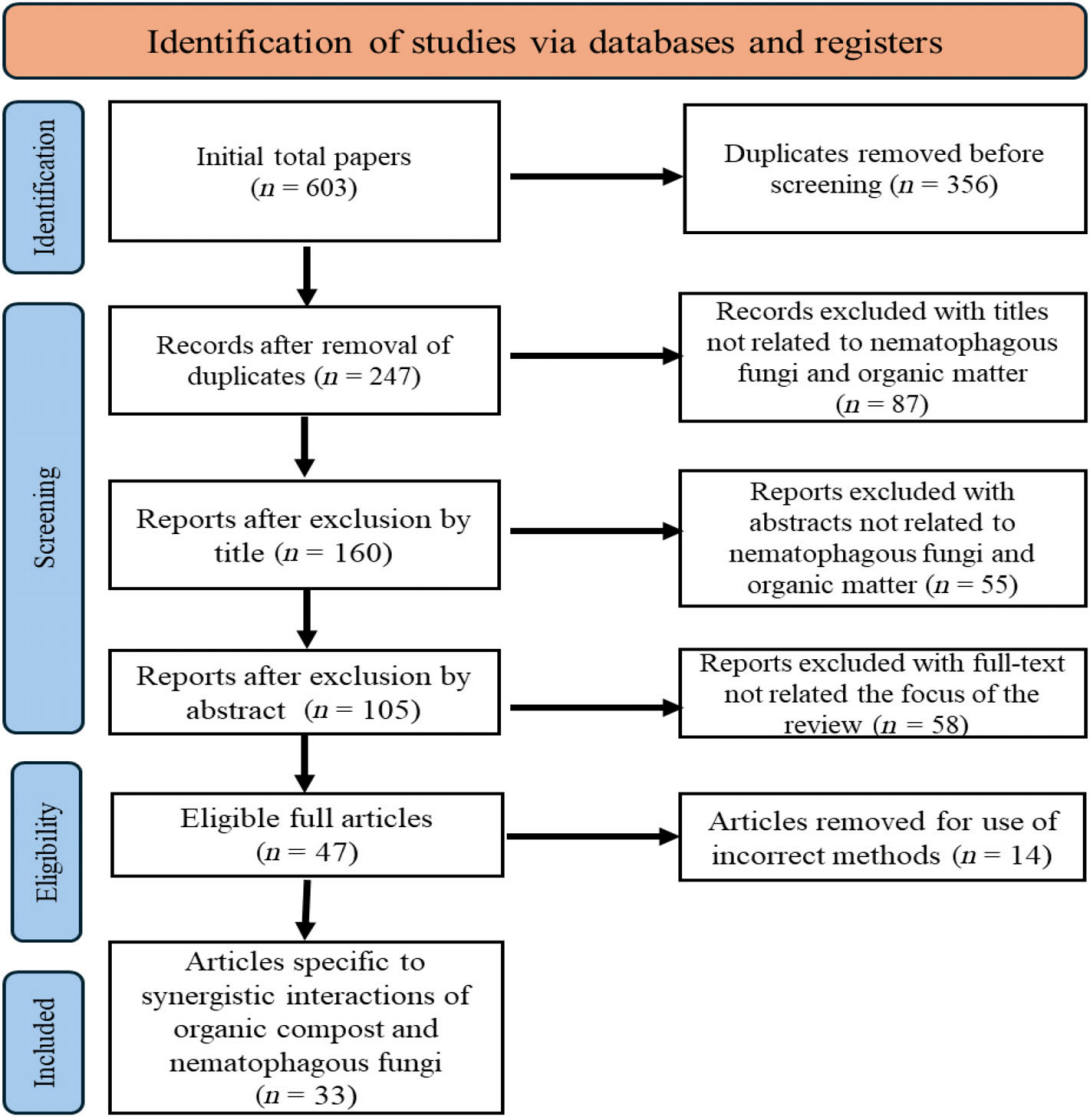
PRISMA flow diagram illustrating the identification, screening, eligibility assessment, and final inclusion of studies evaluating the combined use of nematophagous fungi and organic amendments for the management of plant‐parasitic nematodes.

After a thorough screening procedure based on predetermined inclusion criteria, a total of 33 peer‐reviewed experimental articles were included (Fig. 1). Studies carried out in controlled environments (e.g., greenhouses, net‐houses, or glasshouses) or in the field were eligible, and each one had to have an untreated control. To preserve ecological validity, studies that were only carried out under *in vitro* conditions and lacked control treatments with field relevance were excluded. A structured Microsoft Excel spreadsheet was used for data extraction to ensure uniformity and minimise bias. Key variables recorded from each study included the NF species used, target PPN species, nematode‐susceptible plant species used, the nature and type of organic soil amendments, experimental settings, replications, and study duration, and primary results such as nematode population suppression, egg parasitism rates, and crop performance indicators were extracted. To guarantee accuracy and completeness, each entry was cross‐checked against the original full‐text article. A meta‐analytical technique was not used because of the methodological variability across the investigations, which included differences in fungal strains, amendment formulations, and results measurements. Rather, a mixed‐methods approach that included quantitative and qualitative synthesis was used to analyse the data. Where possible, quantitative outcomes such as percentage nematode suppression, plant growth responses, and infection indices were summarised as ranges and grouped according to fungal species and amendment type to facilitate comparison across studies.

Descriptive summaries of quantitative data, such as percentages of nematode reduction, increases in plant biomass, or infection rates, were categorised according to fungal species and amendment types to find trends in efficacy. Frequency distributions and comparative result ranges were presented wherever feasible to illustrate how well different treatments performed in comparison to one another. The goal of the qualitative synthesis was to find contextual elements and thematic patterns affecting fungal efficacy. These included the kind and stage of organic amendment breakdown, particular fungal strain compatibility with different soil types, and documented synergistic effects between NF and OM. Through recurrent data analysis and theme classification, the lack of specialised statistical or synthesis tools was resolved, and a clear, repeatable presentation of the available evidence was guaranteed. This integrated approach allowed for comprehensive understanding of how soil organic amendments interact with NF under practical conditions, revealing important trends and knowledge gaps relevant to biocontrol‐based nematode management.

## 3 RESULTS

### 3.1 Geographic distribution of reviewed studies

**Figure 2:**
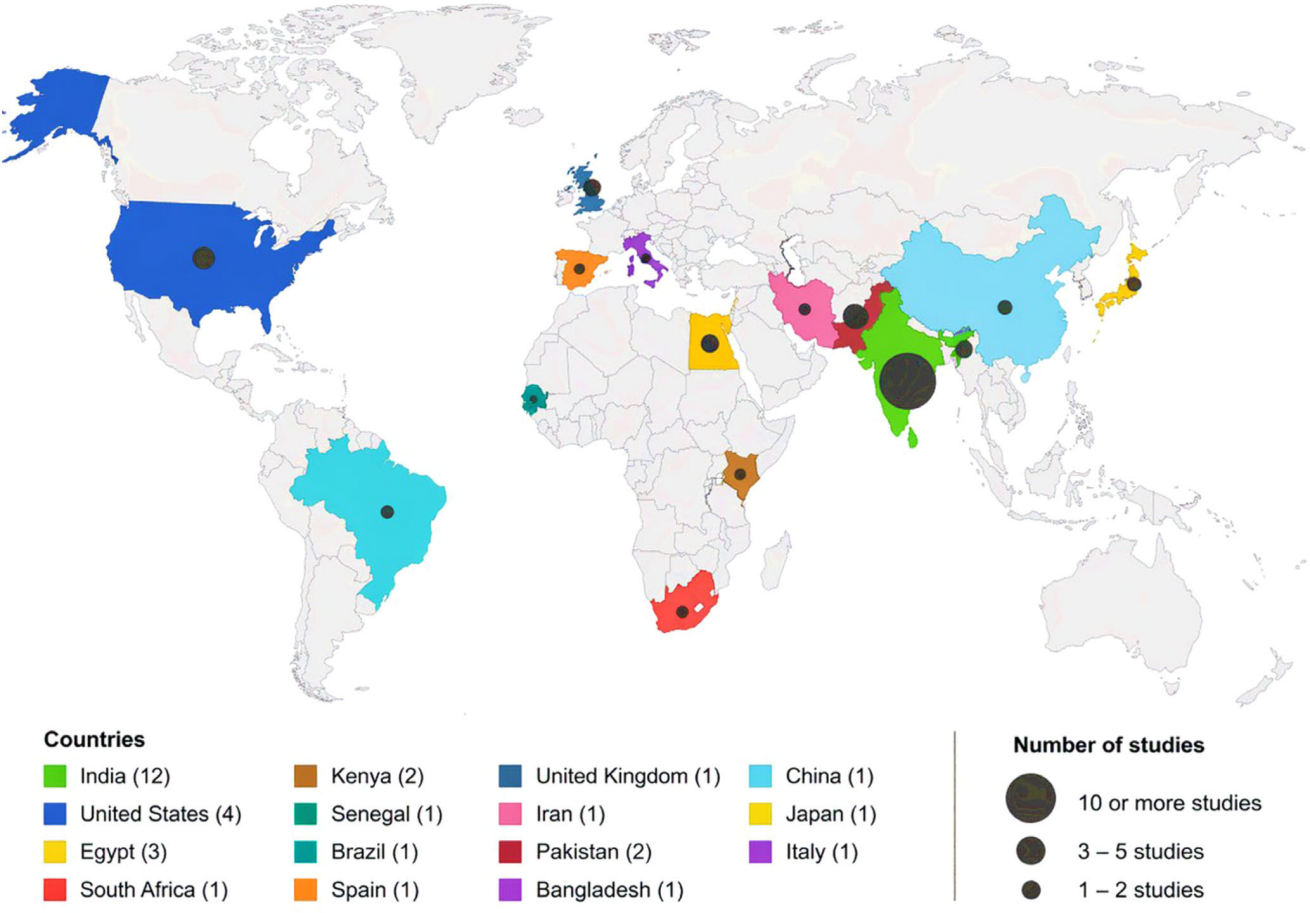
Global distribution of studies on nematophagous fungi combined with organic amendments. Countries are colour‐coded (see legend), and bubble size indicates the number of studies per country.

This systematic review comprised 33 studies covering a wide range of crops, fungal species, geographic regions, and organic amendment types. Most of the research was carried out in India,^7^, ^16^, ^17^, ^18^ Kenya,^10^, ^19^ South Africa,^20^ Egypt,^21^ Iran,^13^ Brazil,^22^ Spain,^23^ Senegal,^24^ China,^25^ the USA,^26^ and England.^27^ This regional concentration, particularly in Asia and parts of Africa, is supported by the observed outcomes, as climatic conditions, soil types, cropping systems, and amendment availability vary across regions. Consequently, the effectiveness of NF–OM interactions reported here may not be directly transferable to underrepresented agroecological zones, highlighting the need for region‐specific validation. The geographic distribution of studies (Fig. 2) reveals a strong concentration in Asia, particularly in India, with comparatively fewer studies conducted in Africa, Europe, and America. This uneven distribution highlights a regional bias in research efforts and suggests that the applicability of findings across diverse agroecological systems remains insufficiently explored.

### 3.2 Overview of study characteristics and trends

While other species, such as *H*. *schachtii*,^28^ *M. hapla*,^26^ and *R*. *reniformis*,^17^ were also reported, *M*. *incognita* and *M. javanica* were the most commonly studied target nematodes.^29^, ^30^ This strong focus on *Meloidogyne* species reflects their global economic importance and adaptability but introduces a taxonomic bias that can limit the applicability of findings to other PPN groups with different life cycles, host interactions, and responses to biocontrol strategies. The most common fungal genera were predatory fungi, including *Arthrobotrys oligospora*,^8^, ^10^, ^24^ egg‐parasitic fungi like *Pochonia chlamydosporia*,^22^, ^23^ and *Purpureocillium lilacinum*.^19^, ^29^

More prevalent NF were secondary metabolite producers, including *Trichoderma viride*, *T. virens*, and *T. harzianum*.^9^, ^13^ Numerous research studies have investigated co‐application with different microbial agents or combinations of fungi.^11^, ^31^ This apparent over‐representation of *Trichoderma* species is associated with their broad‐spectrum activity, ease of mass production, and established use in commercial biocontrol formulations. The host crop that was examined the most was the tomato, which was followed by eggplant, cucumber, eggplant, capsicum, chickpeas, and others. OM including compost, vermicompost, oilseed cakes, neem cakes, poultry manure, chitosan, green manure, press mud, and wasted mushroom substrates were among the organic amendment types that were assessed. In Brazil, for instance,^22^ wine industry wastes were incorporated, and in China,^25^ cow dung and mushroom waste. The length of the studies varied from 4 weeks to 3 years. Glasshouse, greenhouse, screen‐house, net‐house, field, and microplot settings were all used for the experiments. Cobb sieving and decanting, Baermann funnel extraction, sugar flotation, egg staining, root gall indexing, and NaClO‐based root maceration procedures were among the nematode extraction and evaluation methods used.^21^, ^30^, ^32^

**Figure 3:**
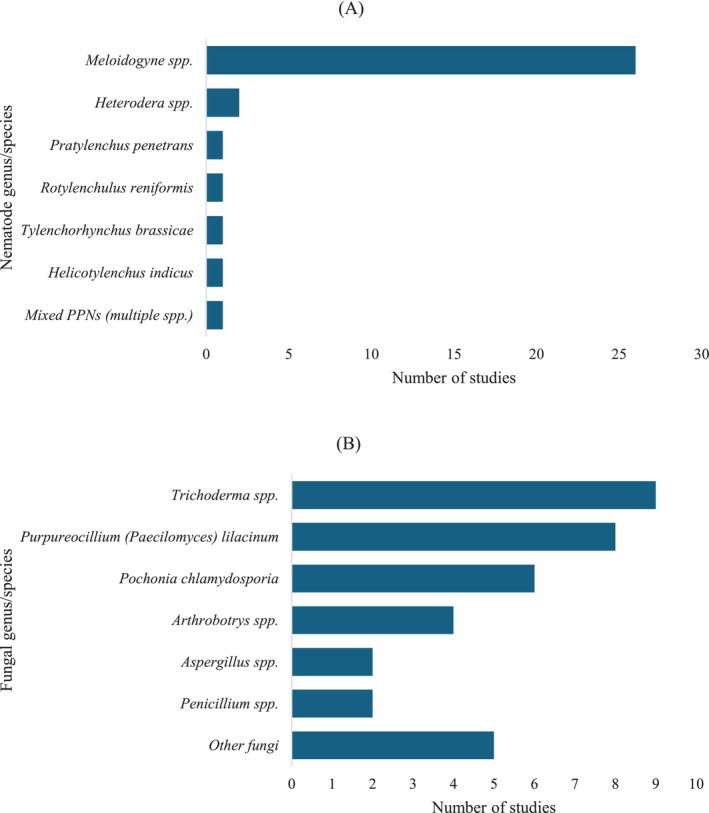
Frequency taxonomic distribution of (A) target plant‐parasitic nematodes taxa and (B) nematophagous fungal genera reported across studies included in this review.

A total of 27 out of 33 studies (81.8%) focused on *M. incognita* and *M. javanica*.^7^, ^29^ There may be a considerable taxonomic bias because other taxa, including *Heterodera*, *Rotylenchulus*, and *Tylenchorhynchus*, were hardly ever examined. This narrowed emphasis could potentially neglect fungi that could be effective against other nematode infestations. The most regularly employed fungi were *T. harzianum* (six studies) and *P. chlamydosporia* (nine studies), which were often chosen because to their easy formulation and known antagonism.^9^, ^23^ Additionally, these species were often evaluated in conjunction with organic inputs as vermicompost, chicken manure, or neem cake. On the other hand, just one or two studies included genera such as *Dactylellina*, *Ficto*r, and *Talaromyces*, suggesting that the variety of NF taxa is understudied. This pattern indicates a strong taxonomic bias towards *Meloidogyne* species, with comparatively limited representation of other economically important nematode genera such as *Heterodera* and *Rotylenchulus*. The distribution of target nematode taxa and NF genera across the included studies is presented in Fig. 3(A), wherein clear taxonomic bias can be observed, with *Meloidogyne* species dominating the dataset, while other nematode genera, such as *Heterodera*, *Pratylenchus*, and *Rotylenchulus*, are considerably less represented. Similarly, a limited number of fungal genera accounted for the majority of studies, with *Trichoderma*, *P. lilacinum*, and *P. chlamydosporia* being most frequently investigated (Fig. 3(B)).

**Figure 4:**
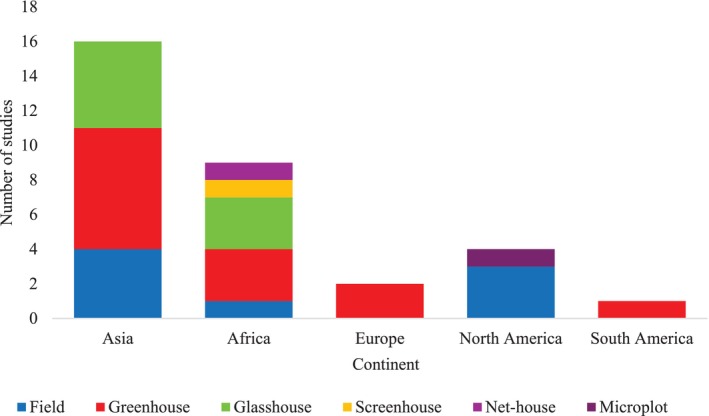
Distribution of experimental conditions across geographic regions in studies evaluating the combined use of nematophagous fungi with organic amendments against plant‐parasitic nematodes.

The predominance of specific organic amendments (e.g., vermicompost and poultry manure) suggests a concentration of research on readily available and nutrient‐rich inputs, with comparatively fewer studies exploring alternative or underutilised amendment types. Vermicompost, poultry manure, and neem‐based products were used in 19 out of 33 studies (57.6%), making them the most commonly applied organic amendments.^33^ In contrast, other amendment types (e.g., green manure, biochar, or crop residues) were less frequently represented, indicating a comparatively limited exploration of their potential in NF‐based nematode management. Press mud,^34^ waste mushroom substrates,^25^ and wine‐industry byproducts^22^ were less often used amendments that could have new advantages but need further research. Relatively few studies^17^, ^18^ lasted more than one growing season, and the majority were carried out over 45–90 days. These brief periods are generally sufficient for evaluating initial efficacy but do not capture longer‐term effects on soil suppressiveness, nematode population recovery, and fungal persistence, which limits assessment of the durability and stability of NF–OM interactions over time. About 60% of the research was carried out in controlled settings, including glasshouses, screen houses, and greenhouses^8^, ^32^ (Fig. 4).

### 3.3 Synergistic suppression of PPNs by NF and OM

With regards to OM incorporation, Table 2 shows that *Trichoderma*, *Pochonia*, and *Purpureocillium* were the most investigated NF taxa.^18^, ^29^ In contrast to nematode‐inoculated controls, plants treated with compost or *Arthrobotrys oligospora* alone showed notable increases in growth and yield according to comparative evaluations like the one conducted by Danish *et al*.^8^ However, when compost and fungus were administered together, *M*. *incognita* was substantially inhibited, suggesting a synergistic effect. The growth and reproductive rate of *M. mayaguensis* in tomatoes were considerably decreased by amendments including *Eucalyptus camaldulensis*, *Azadirachta indica*, and *Acacia holosericea* leaves, according to a study by Duponnois *et al*.^24^ These organic inputs alone decreased nematode populations at modest application rates, and improved suppression was shown when paired with *A. oligospora*. Particularly in net‐house and field settings, the incorporation of neem cake and poultry manure with *P. lilacinus* also led to significant decreases in nematode and root galling populations in *Capsicum annuum*.^29^ However, not all studies reported consistent or enhanced outcomes, with some indicating limited or context‐dependent effects of OM–NF combinations depending on amendment type, application rate, and environmental conditions.

**Table 2:** Summary of experimental studies investigating the use of nematophagous fungi in combination with organic soil amendments for the control of plant‐parasitic nematodes

Fungal species	Target nematode	Crop	Soil amendment	Study duration	Observations	Condition	Quality score	Location	Source
*Arthrobotrys oligospora*	*Meloidogyne mayaguensis*	Tomato	Powdered leaves of sorghum, Australian pine, neem, river red gum, candelabra wattle, brown salwood	1 month	Glasshouse	While leaf amendments suppressed tomato growth, the combination with *A. oligospora* improved plant vigour and enhanced the antagonistic activity of *A. oligospora*	3	Senegal	24
*A*. *oligospora*	*Meloidogyne* spp.	Tomato	Cattle and poultry manure	7 weeks	Screen‐house	Amending soil with organic materials significantly stimulated the growth of *A*. *oligospora*, effectively reducing *Meloidogyne* populations	3	Kenya	10
*A*. *oligospora*	*M*. *incognita*	Ajwain	Composted organic matter	4 months	Greenhouse	Plants treated with *A. oligospora* alone or in combination with urea and composted organic manure had significant decrease in number of gall (86.89%), size of gall (76.52%), number of egg masses (88.86%), and disease index and improved growth, yield and biochemical characteristics when compared to untreated control	5	India	8
*A. oligospora*, *A. eudermata*, *Dactylellina haptotyla*, *D. ellipsospora*	*Heterodera schachtii*	Vineyard	Grape leaves	2 years	Field	Incorporating leaves enhanced *D. haptotyla* population density and trapping activity in 2001, but not in 2000. In the second vineyard plot, leaves enhanced the population density but not the activity of resident trapping fungi (*A. oligospora* and *A. eudermata*)	4	San Joaquin	28
*Aspergillus* spp., *Alternaria alternate*, *Paecilomyces lilacinus, Verticillium chlamydosporium*, *Fusarium oxysporum*	*M*. *incognita*	Tomato, eggplant	Chicken manure, eucalyptus leaves/stems powder	40 days	Greenhouse	Chicken manure alone gave the highest gall reduction (59.02%), followed by eucalyptus leaves and stems dry powder (38.37%), and the mixture of chicken and eucalyptus (39.33%)	4	Egypt	35
*D. candidum*	Plant‐parasitic nematodes	Vineyard	Alfalfa leaves	84 days	Field	Fungivorous nematodes were abundant in alfalfa‐amended soil and their potential to suppress trapping fungi requires more research	3	USA	36
*Aspergillus terreus*, *Talaromyces minioluteus*, *T. sayulitensis*, *Trichoderma ghanense*, *T. viride*	*M. enterolobii*	Dry bean	Compost	45 days	Net‐house	Incorporating organic compost enhanced nematode eggs and juvenile antagonism by 63–95%	4	South Africa	20
*A*. *niger*, *Penicillium digitatum*, *T*. *viride*	*M*. *incognita*, *Rotylenchulus reniformis*, *Tylenchorhynchus brassicae*, *Helicotylenchus indicus*	Chickpea	Mexican poppy, apple of Sodom, Yellow‐berried nightshade, water hyacinth	3 years	Field	Soil botanicals significantly reduced multiple plant‐parasitic nematodes, increased beneficial saprophytic fungi, and improved chickpea growth, physiology, and nodulation	5	India	17
*A. niger*, *Paecilomyces lilacinus, Penicillium chrysogenum*	*M*. *incognita*	Tomato	Cattle manure	3 months	Glasshouse	The highest increase (79%) in the growth of nematode‐inoculated plants was observed when *P. putida* was used with cattle manure, followed by use of *P. lilacinus* + cattle manure	4	Japan	37
*Fictor composticola*	*M. incognita*	Cucumber	Neem cake, poultry manure, neem leaves	45 days	Glasshouse	All combinations of organic amendments were found significantly superior over inoculated control in reducing nematode populations and in improving plant growth	4	India	33
*Paecilomyces marquandii*	*M. hapla*	Lettuce	Chitin, wheat mash, brewery compost	7 weeks	Field microplot	While all three amendments were effective against *M. hapla* and reduced root galling, wheat mash amendment increased lettuce head weight; however no antagonist × amendment interaction was detected	4	USA	26
*Pasteuria penetrans*	*M. incognita*	Eggplant	Sawdust, Kanair (leaves)	6 weeks	Greenhouse	Highest *M. incognita* suppression (61%) in root galling was recorded in pots treated with Azadirachtin + *P. penetrans* followed by Abamectin + *P. penetrans* (52%), Abamectin + *P. penetrans* (48%), Lorsban + *P. penetrans* (42%), Azadirachtin (36%), and Lorsban (21%) compared with unamended control	5	Pakistan	38
*Pochonia chlamydosporia*	*M. javanica*, *M. incognita*	Tomato	Chitosan	2 months	Greenhouse	Chitosan irrigation increased dry shoot, fresh, and root weight of tomato plants inoculated with *M. javanica*, as well as root length in plants inoculated with *P. chlamydosporia*	4	Spain	23
*P. lilacinus*	*M*. *javanica*	Tomato	Solid by‐product of the wine industry	60 days	Greenhouse	Application of *P. lilacinus* in tomato resulted in ~85% infection of *Meloidogyne* egg masses and improved plant growth compared with nematode‐only treatments	4	Brazil	22
*P. chlamydosporia*	*M. incognita*	Chickpea	Freshly chopped leaves of billy goat weed and water hyacinth	60 days	Glasshouse	The combined application of *P. chlamydosporia* + billy goat weed was found highly effective, and water hyacinth alone was least effective	4	India	7
*P*. *chlamydosporia*	*M. javanica*	Eggplant	Oil seed cakes of different plant species, i.e., neem, mustard, cotton	60 days	Glasshouse	Among the oil cakes, neem cake was more effective than the other individual treatments in managing *M. javanica*, whereas among the combined treatments neem cake + *P. chlamydosporia* was the most effective, followed by mustard cake + *P. chlamydosporia* and cotton cake + *P. chlamydosporia*	4	India	39
*P. chlamydosporia*, *P. lilacinus*	*Meloidogyne* spp., *Heterodera* spp.	Tomato	Maize straw, straw, rattlepods cabbage leaves	4 weeks	Greenhouse	Maize straw was found to be a more effective organic amendment which favoured the activity of *P. chlamydosporia* and *P. lilacinus*, while *P. chlamydosporia* was found to exhibit a high percentage parasitism against *Meloidogyne* spp.	3	England	27
*P. chlamydosporia*, *Purpureocillium lilacinus*, *T. harzianum*	*M. incognita*	Eggplant	Dry neem	4 months	Field	Combining biocontrol fungi with dry neem leaves effectively managed *M. incognita* by reducing population density and galling index, while improving plant growth parameters	4	India	11
*P*. *chlamydosporia*, *T*. *viride*	*M. incognita*	Kidney bean	Urea	60 days	Greenhouse	Combining *P. chlamydosporia* or *T. viride* with urea significantly managed *M. incognita* and increased bean growth	4	India	30
*P. lilacinus*	*Meloidogyne*	Pepper	Neem cake, farmyard manure	70 days	Net‐house and field	Split application of *P. lilacinus*‐enriched neem cake (1 t/ha) plus farmyard manure (2.5 t/ha) reduced root galling by 44.52% (net house) and 50.60% (field); single application reduced galling by 41.60% and 45.51%, respectively.	5	India	29
*P. lilacinus*	*M. incognita*	Tomato	Marigold, castor oil plant, thorn apple	60 days	Greenhouse	Galling intensity and the population of juveniles were significantly lower in soils amended with organic matter	4	Kenya	19
*P*. *lilacinus*, *Rhizoglomus fasciculatum*	*M. incognita*	Pepper	Organic manure, green manure	60 days	Greenhouse	Green manure along with *R. fasciculatum* and *P. lilacinus* showed a significant increase in parasitising *M. incognita* eggs, with maximum effect detected on dual inoculation	4	India	12
*P. lilacinus*, *Cladosporium oxysporum*	*M. incognita*	Eggplant	Oil cakes (groundnut, neem, linseed, castor, mahua)	–	Glasshouse	Integration of *P. lilacinus* with groundnut cake produced the greatest plant growth improvement and *M. javanica* suppression, followed by neem, linseed, castor, and mahua cakes; *C. oxysporum* performed best with neem cake combinations	3	India	40
*Pleurotus eryngii*	*M. incognita*	Tobacco	Spent mushroom substrates, cow dung	45 days	Greenhouse	Soil amended with vermicompost showed 61% control efficiency against nematode disease caused by *M. incognita* on tobacco, which was significantly higher than that of the normal compost (24%)	4	China	25
*Purpureocillium lilacinum*	*Pratylenchus penetrans*	Potato	Raw poultry manure, other manure‐based amendments	Two field seasons	Field	Combined application of manure‐based amendments and *P. lilacinum* effectively maintained low *P. penetrans* populations in both soil and roots	5	USA	61
*T. harzianum*	*M. incognita*	Scurf pea	Press mud	–	Glasshouse	Combined application of *T. harzianum* and press mud showed significant reduction in the number of egg masses, nematode population, and root‐knot index	3	India	34
*T*. *harzianum*, *T. viride*, *T. virens*	*M. incognita*	Pea	Seeds of fennel and caraway, powdered leaves of basil	3 months	Screen‐house	Combination of fungi and organic amendments caused higher average total percentages of *M. incognita* reduction than those achieved by single treatments	4	Egypt	31
*T. harzianum*	*M. incognita*	Onion	Poultry refuse, mustard oilcake, rice bran, saw dust	3 years	Field	*Trichoderma* compost and poultry refuse showed the highest *M. incognita* reduction and significantly increased the growth of onion with the highest yield	5	Bangladesh	9
*T. harzianum*	*M. javanica*	Chickpea	Vermicompost, neem cake	2 years	Field	Organic amendments and biocontrol agent reduced nematode population considerably in comparison to control treatment, with results showing reduced root galling on all the treatments compared with the control	5	India	18
*T. harzianum*	*M. incognita*	Tomato	Organic matter	1 year	Greenhouse	Integration with *T. harzianum* and organic fertiliser significantly reduced *M. incognita* and resulted in the greatest increases of plant growth and yield	4	Italy	32
*T. harzianum, P. lilacinus*	*M. javanica*	Okra	Oil cakes (e.g. neem cake, cotton seed cake)	6 weeks	Greenhouse	Combined use of fungi and oil cakes significantly reduced *M. javanica* infection while exhibiting the greatest height and fresh shoot weight	4	Pakistan	41
*T. virens, Pseudomonas fluorescens*	*M. javanica*	Tomato	Oak tree debris	3 months	Greenhouse	Treatments producing the greatest effects: *T. virens* reduced root galling by 56.3%, while root dry weight increased by 68.2% with *P. fluorescens* and 56.1% with *T. virens* compared with unamended controls	4	Iran	13
*Verticillium chlamydosporium*	*M. incognita*	Cucumber	Animal compost	2 months	Greenhouse	Maximum reduction in gall formation, female numbers, egg‐mass production, developmental stages, and final population of juveniles in soil was observed with *V. chlamydosporium* + *P. luminescens* + animal compost treatment combinations, compared with the control and other treatments	4	Egypt	21

Even though *Trichoderma* is a common biocontrol agent,^9^, ^13^ it does not infect nematodes directly, in contrast to *Arthrobotrys* species, which create trapping structures to feed on juveniles, or *P*. *chlamydosporia* and *P*. *lilacinum*, which parasitise nematode eggs. However, it has been demonstrated that *T. harzianum* increases plant vigour and lessens galling severity when mixed with nutrient‐rich amendments such press mud, mustard oil cake, or vermicompost.^18^, ^34^ It is noteworthy that research that combined more than one fungal species or paired fungi with chemically active additives (such as chitosan or mushroom substrates) showed improved nematode suppression that went beyond additive effects. In tomato experiments, for instance, *P. chlamydosporia* and chitosan led to lower egg mass density and higher parasitism rates.^23^ According to Yang *et al*.,^25^ *Pleurotus eryngii* treated with cow dung and mushroom waste considerably reduced the infestation of *M. incognita* in tobacco.

## 4 DISCUSSION

### 4.1 Synergistic relations between NF and OM

Through reciprocal advantages, the cooperative interaction between NF and OM improves soil health and PPN biological control. OM supports NF through interconnected mechanisms, including improved nutrient availability, enhanced soil microhabitats, stimulation of fungal metabolic activity, and buffering against environmental stress. On the other hand, through increasing the breakdown of complex organic compounds, stabilising OM within soil aggregates, improving nutrient mobilisation/retention, and sustaining a variety of microbial communities, NF in turn contribute to the dynamics of OM.

#### 4.1.1 How OM benefits NF

The enhanced activity of NF in OM‐amended soils is largely driven by improved nutrient availability and ecological conditions. Empirical studies consistently show that the integration of OM with fungal biocontrol agents improves nematode suppression, plant performance, and fungal persistence compared to individual applications. These outcomes are primarily driven by improved nutrient availability, favourable microhabitat conditions, and stimulation of fungal metabolic activity. According to Tang *et al*.,^42^ OM supplies readily available carbon and nitrogen compounds that support NF growth, for instance *A*. *oligospora*, *P*. *lilacinum*, and *P*. *chlamydosporia* have shown increased conidiation and predatory activity in the presence of compost, farmyard manure, and green waste residue.^8^, ^18^, ^22^ These fungi responded particularly well to OM rich in labile carbon such as vermicompost and poultry manure, which stimulate microbial mineralisation processes on N, P, and other micronutrients. In a study by Yu *et al*.,^43^ compared to *P. lilacinus* grown on potato dextrose agar, the nematocidal capacity of *P. lilacinus* cultivated on food waste was 10.8% and 27% greater, respectively. Overall, nutrient‐rich OM inputs enhance both the activity and persistence of NF across systems. Moreover, OM amendments help stabilise nutrient availability during stress events, minimising nutrient leaching and maintaining a continuous supply of essential elements for fungal growth and enzyme production.^44^ These conditions not only preserve NF viability but also enhance their capacity to resume parasitism activity rapidly when stress conditions subside.

Beyond improving nutrient availability, OM plays a central role in improving soil microhabitats, which directly benefits survival, colonisation, and predatory efficiency in NF. By enhancing soil structure, OM increases porosity and aggregate stability, which in turn promotes better aeration and water retention.^45^ These conditions create a stable microhabitat that supports the proliferation of NF hyphae and formation of trapping structures. For example, Escudero *et al*.^23^ demonstrated that the combination of *P. chlamydosporia* with chitosan significantly reduced root galling and nematode proliferation compared to individual treatments, while also improving plant biomass. Similarly, Parihar *et al*.^39^ reported enhanced egg parasitism rates exceeding 50% when *P. chlamydosporia* was combined with neem cake, alongside measurable yield improvements. Fungi such as *A*. *oligospora*, *D*. *brochopaga*, and *Monacrosporium cionopahum* rely on thin water films to maintain hyphal activity and nematode‐trap adhesion, conditions that are improved in OM‐rich soils, including compost and leaf litter.^46^ Studies have shown that in OM‐amended soils, predatory fungi exhibit significantly higher trap density nematode capture rates compared to a non‐amended control. These findings suggest that OM not only enhances resource availability but also creates a dynamic, biologically active niche that sustains NF under both field and controlled environments. Liang *et al*.^47^ further established that these stable microsites also support interactions with beneficial microbial consortia, including bacteria, indirectly assisting fungal colonisation.

The induction of NF activity in OM‐amended soils is a key mechanism by which NF are activated and maintained in agroecosystems. OM such as compost, manure, and vermicompost release a broad spectrum of soluble organic compounds.^48^ These compounds serve as chemical signals and substrates that stimulate metabolic activation and hyphal growth in fungi such as *A*. *oligospora*, *P*. *lilacinum*, and *P*. *chlamydosporia*. Experimental evidence further indicates that organic amendments enhance fungal predatory efficiency through improved soil structure. Liu *et al*.^49^ reported a greater density of adhesive networks in *A. oligospora* when grown in straw‐amended soils, resulting in over 60% higher nematode‐trapping efficiency. Under field conditions, Jaffee^28^ observed increased nematode mortality in organically amended soils, highlighting the ecological relevance of these interactions. Trap formation is often inducible rather than constitutive, with organic cues and nematode presence acting as dual triggers that shift fungi from saprophytic to predatory behaviour.^48^ Certain compounds in OM, particularly low‐molecular‐weight compounds, have been found to up‐regulate genes involved in trap development, conidiation, and protease secretion. For example, incorporation of plant material increased the expression of subtilisin‐like serine proteases, which are critical for nematode cuticle degradation.^50^ According to Timper,^51^ in OM‐amended soils, NF trap density and enzymatic activity peak days after application, aligning with the early stages of OM decomposition and microbial succession.

The incorporation of OM reduces fluctuations in soils temperature and moisture, buffering NF against abiotic stress.^52^ Biochar and manure‐based amendments, for example, help sustain microsites with favourable moisture conditions even under intermittent drought, extending fungal activity periods. Soils enriched with OM such as compost, biochar, or manure exhibit improved water‐holding capacity, aggregate stability, and thermal regulation, all of which help mitigate stress conditions such as drought, extreme temperatures, or nutrient fluctuations.^53^ Organic residues slow evaporation and maintain thin films of moisture around aggregates, which are essential to fungal hyphal function and nematode‐trap adhesion. In addition to physical buffering, OM also contributes to biochemical stress tolerance. Humic substances and phenolic compounds present in compost and manure have been linked to antioxidant effects and protective enzyme activation in fungi, which reduce oxidative damage under abiotic stress.^54^ The persistence of fungal biocontrol agents is also enhanced in organically amended systems. Khan *et al*.^11^ showed that the combined application of *P. lilacinum* with vermicompost resulted in reduced galling indices and improved plant biomass, while Li *et al*.^55^ demonstrated sustained fungal population densities and prolonged suppression of nematode populations in amended soils. Field and greenhouse studies have demonstrated that OM applications, such as 40 t/ha of compost or 2–5% of biochar, improve fungal persistence by up to 40–60% under drought or thermal stress conditions compared to non‐amended soils.^56^

The effectiveness of these interactions is strongly influenced by the type of organic amendment applied. For instance, neem‐based amendments provide both a nutrient source and direct nematicidal compounds such as azadirachtin, while poultry manure and oilseed cakes supply high nitrogen content that promotes rapid microbial activity. Composted materials, on the other hand, improve soil structure and microbial diversity, thereby creating favourable conditions for fungal colonisation and activity.

#### 4.1.2 How NF improve soil OM

Beyond their role in PPNs suppression, NF are also associated with contributing to OM dynamics, with accelerated decomposition representing one pathway through which these effects are realised. During their predatory phase, fungi such as *A*. *oligospora* and *P*. *chlamydosporia* produce a suite of extracellular enzymes, particularly proteases, chitinases, lipases, and cellulases, that not only degrade nematode tissues but also act on complex organic substrates in the soil.^57^ When these fungi colonise OM such as compost, vermicompost, or manure, the enzymatic mechanisms activated for PPN degradation is similarly utilised for the breakdown of proteinaceous and chitin‐rich organic substrates, accelerating mineralisation processes and enhancing the release of bioavailable nitrogen and carbon compounds. Furthermore, nematode predation itself is linked to decomposition indirectly by converting nematode biomass into simpler compounds, which are rapidly assimilated by soil microbes or reabsorbed by NF. For example, *P*. *chlamydosporia* infects nematode eggs and degrades their gelatinous matrix and chitin‐rich shells, releasing amino sugars and nitrogenous compounds that could enrich the rhizosphere microbial community.^23^ When NF are introduced into soils containing OM such as manure, green waste, plant residues, their dual capacity to act as biocontrol agents and decomposers becomes evident, as shown by increased enzymatic activity and faster carbon turnover rates.^22^ This dual functionality is associated with enhancing the microbial efficiency of decomposition pathways, positioning NF as potential contributors to both pest control and soil OM dynamics in agroecosystems.

More than accelerating decomposition and biocontrol, NF may also contribute to the stabilisation of OM in soils, a function that enhances long‐term soil carbon storage and structural integrity.^58^ As fungi such as *A*. *oligospora* and *P*. *chlamydosporia* metabolise organic inputs, they contribute to the formation of microbial necromass and extracellular polysaccharides that become physically protected within soil aggregates. This necromass, often derived from fungal hyphae and enzymatically processed residues, binds with mineral particles and humic substances, slowing further degradation and enhancing the persistence of organic carbon in the soil matrix. Studies have shown that in soil amended with OM, the presence of NF is associated with increased aggregation and increased proportion of organic carbon in the particulate OM and mineral‐associated OM fractions. The mechanisms involved include both biochemical transformations, via conversion of labile organic substrates into more recalcitrant microbial products, and biophysical protection, where fungal hyphae act as a structural scaffold that enmeshes organic residues and soil particles, promoting aggregate stability.

NF has also been reported to play a role in enhancing nutrient mobilisation and retention in organically amended soils. Through the production of extracellular enzymes such as proteases, chitinases, and phosphates, NF such as *A*. *oligospora* and *P*. *chlamydosporia* contribute to the breakdown of organic and biological residues, including nematode carcasses, egg masses, and protein‐rich organic amendments.^58^ This enzymatic activity can liberate key nutrients, particularly N, P, Zn, and Fe, from complex organic forms, increasing their availability to plants and other soil microbes. For example, *P. chlamydosporia* has been shown to release orthophosphate during nematode degradation, while *A. oligospora* enhances ammonium‐N availability during compost mineralisation.^58^ Importantly, NF not only mobilise nutrients, but may also contribute to their retention in soil through direct assimilation into fungal biomass and the formation of microbial residues that bind to mineral surfaces and soil aggregates. These stabilised organic–mineral complexes reduce nutrient leaching and support longer‐term nutrient cycling.

NF can contribute to the enrichment and structuring of the soil microbial food web, creating ripple effects that enhance OM turnover and stabilisation.^59^ By parasitising and preying on PPNs, NF such as *P*. *chlamydosporia* introduce biologically derived organic inputs into the soil system in the form of decomposed nematode biomass and fungal necromass. These inputs are rapidly assimilated by bacteria, actinomyces, and saprophytic fungi, which may stimulate microbial succession and promote trophic complexity in the soil food web.^36^ This microbial enrichment is particularly evident in organically amended soils, where the combined presence of decomposing OM and active NF supports broader diversity and activity of decomposers, secondary consumers, and beneficial rhizosphere organisms. The increased microbial activity has potential to accelerate the transformation of labile organic compounds into microbial products, such as extracellular polysaccharides and necromass, that are more readily stabilised in soil aggregates and mineral surfaces. In this way, NF indirectly promote the microbial‐mediated stabilisation of OM fractions, reinforcing their potential role in linking biological control with soil OM dynamics.^60^

## 5 RESEARCH GAPS AND PRACTICAL RECOMMENDATIONS

The integration of NF with OM amendments presents a biologically enriched, ecologically sustainable strategy for suppressing PPNs. However, this approach remains underexplored, with several critical research gaps that warrant attention. While individual roles of NF in nematode suppression are extensively documented, the mechanistic basis of their interaction with OM remains poorly resolved. The molecular signalling mechanisms that govern fungal activation in response to OM remain underexplored. Although trap‐inducing cues such as volatile organic compounds and nitrogenous breakdown products have been suggested, the exact chemical signals released during OM decomposition and the fungal receptors they target remain unclear. A related gap lies in microbial interactions within these systems. Fungi rarely operate alone; rather, they are part of complex microbial consortia where bacteria and other microbes may facilitate or inhibit fungal activity. Nevertheless, the identities and ecological roles of these microbial partners under organic amendment regimes remain poorly mapped, and methods such as stable isotope probing and co‐occurrence network analysis remain underutilised.

Moreover, the spatiotemporal dynamics of this synergy under field conditions are not well understood. Several studies are conducted under controlled conditions, limiting understanding of how soil texture, amendment type, and application frequency influence NF persistence and activity under field conditions. Another critical gap is the lack of amendment‐specific and dose–response data. Relatively few comparative studies have examined how different organic inputs, such as compost, green manure, or biochar, affect NF gene expression, enzyme activity, and PPN suppression efficacy. Moreover, long‐term ecological consequences of repeated OM application on fungal population, nematode community structure, and soil organic carbon stability are poorly quantified. Long‐term field trials that integrate microbial functional diversity, PPNs suppression metrics, and soil health indicators are needed to assess the persistence and resilience of these interactions across cropping cycles. Addressing these gaps is essential for translating NF–OM interactions into scalable biocontrol strategies. Omics‐based approaches (metagenomics, transcriptomics, metabolomics) can reveal the pathways involved in tripartite interactions between fungi, OM, and PPNs.

Another critical and often overlooked gap in the study of NF and OM synergy is the limited integration of environmental variability with economic performance metrics, including yield impact assessment and cost‐effectiveness comparisons with synthetic nematicides. Current studies provide limited insight into how variations in soil texture, OM composition, climate, and crop type influence biocontrol outcomes. This lack of ecological realism undermines the ability to conduct robust cost–benefit analyses, which are essential for determining the scalability and profitability of these strategies across diverse farming systems. Although fungi such as *A*. *oligospora* and *P*. *chlamydosporia* demonstrate strong PPN suppression in experimental settings, few studies track their impact on crop yields, input costs, labour requirements, or long‐term returns under variable field conditions. Moreover, most economic evaluations fail to account for non‐monetised ecosystem benefits such as improved soil structure, enhanced microbial diversity, and reduced ecotoxicity, all of which contribute to agroecosystem resilience but are often excluded from traditional financial models. This evidence gap has direct policy implications, limiting the development of incentive structures, subsidies, and extension guidelines for fungal‐based biocontrol. Future research should prioritise interdisciplinary trials under variable field conditions and incorporate economic analyses that account for both yield and ecological benefits.

## 6 CONCLUSION

This review highlights a biologically relevant interaction between NF and OM, which may function as a reinforcing system for suppressing PPNs while also contributing to improved soil health and ecological balance. OM improves nutrient availability, microhabitats, and stress buffering for NF, while these fungi reciprocate by contributing to OM dynamics through decomposition, stabilisation, and nutrient cycling. Together, these interactions can facilitate nematode suppression and nutrient cycling, contributing to the development of more suppressive soil environments driven by biological processes rather than synthetic inputs. The implications of this partnership extend beyond crop protection. This NF–OM interaction is consistent with the principles of climate‐smart agriculture, with potential benefits for productivity through improved PPN suppression and nutrient availability, enhanced soil resilience, and reduced reliance on synthetic nematicides. Moreover, it is also relevant to several sustainable development goals including SDG 2 ‘Zero Hunger’, SDG 12 ‘Responsible Consumption and Production’, SDG 13 ‘Climate Action’, and SDG 15 ‘Life on Land’. Realising this potential will require coordinated efforts in research, microbial biocontrol innovation, and farmer capacity development. In this way, the interaction between NF and OM represents a promising component of regenerative agriculture and climate‐resilient food systems.

## CONFLICT OF INTEREST

The authors declare no conflicts of interest.

## Data Availability

Data sharing not applicable to this article as no datasets were generated or analysed during the current study.
